# How freshmen perceive Environmental Education (EE) and Education for Sustainable Development (ESD)

**DOI:** 10.1371/journal.pone.0208910

**Published:** 2019-01-14

**Authors:** Michaela Maurer, Franz Xaver Bogner

**Affiliations:** Centre of Math & Science Education, Department for Biology Education, University of Bayreuth, Bayreuth, Germany; The Hague University of Applied Science, NETHERLANDS

## Abstract

Concepts of 464 university freshmen towards Environmental Education (EE) and Education for Sustainable Development (ESD) were analyzed. Responses were classified into seven main categories: ‘ecological aspects’, ‘ecological problems’, ‘economical aspects’, ‘social aspects’, ‘environmental attitudes’, ‘environmental behavior’ and ‘education’. Analyses of sustainability concepts show a large discrepancy between EE and ESD, whereby the latter includes an additional sub-group: ‘the next generation aspect’. Labeling individual sources of EE in a retrospective assessment identified the family as the most important source of knowledge, followed by media, school and outreach. Further differences were detected between students’ self-perception and their ideal conception of environmental behavior, by using the scale Inclusion of Nature in Self (INS). Only some EE statements produced higher (unfulfilled) expectations ‘economic aspects’, ‘environmental behavior’ and ‘ecological problems’. In contrast fewer (unfulfilled) expectations were observed in the categories of ‘education’ and ‘ecological aspects’.

## Introduction

### Overview of Environmental Education history

In addition to Environmental Education (EE), the term Education for Sustainable Development (ESD) has been in use for several decades. Do the concepts behind EE and ESD overlap? Initial approaches to natural phenomena in EE go back to early European Educational Reformers (*e*.*g*. Comenius, Rousseau, Pestalozzi, Goethe and Humboldt), long before ‘EE’ was defined, and before the attempt was made to integrate EE approaches into a general concept of education [1]. At the UCN/UNESCO ‘International Working Meeting on Environmental Education’ (USA, 1970), the ‘original definition’ of EE was “… the process of recognizing values and clarifying concepts to develop skills and attitudes necessary to understand and appreciate the inter-relatedness among man, his culture, and his biophysical surroundings. EE also entails practice in decision-making and self-formulation of a code of behavior about issues concerning environmental quality” [2]. A few years later, the term EE was recognized at the UN Conference on Human Environment in Stockholm [3]. The earliest environmental movements triggered the publication of Carson's book: ‘Silent Spring’, in which she claimed that DDT and other pesticides harm the environment [4] (DDT was found in *e*.*g*. Adèlie penguins and Weddell seals) [5]. Years later environmental movements used this claim to enforce a global ban of DDT. Due to the environmental problems of the 20^th^ century, such as ‘acid rain’ (*e*.*g*. [6]), ‘air pollution’(*e*.*g*. [7]) or ‘ozone layer decline’(*e*.*g*. [8]), the global population developed an increasing environmental awareness, compatible with the EE goals concerning ‘attitudes’, ‘motivations’ and ‘commitment to work individually towards solutions of current problems’ [3, 9, 10, 11]. These goals were reinforced after the Belgrade Charter [9] and expanded in the Tbilisi Declaration in the late 70s [10]. In the 90s, the Rio-Conference defined EE in a broader sense, by developing a global action plan (‘Agenda 21’) with regard to sustainable development (SD) [11]. Although the term ‘SD’ originated in the book *Silvicultura oeconomica* (1713) by Carlowitz, that focused only on ‘forestry’ [12], today the term ESD includes ‘local’, ‘national’ and even ‘global actions’, which deal with present and future aspects of SD as a new guide for ‘lifelong development competencies’ [11]. ESD is a combination of three aspects: environmental (ecological), economic (including poverty reduction, corporate responsibility and accountability of society) and social (including employment, human rights, gender equity, peace and human security) aspects [13, 14, 15]. In graphic representations, they are often illustrated a same-sized circles with a circle labelled “human well-being” in the center representing the quality of life [16]. To the present day, the relationship between EE and ESD has been controversial: some authors consider ESD as the most effective approach to deal with current problems, as ESD may best meet the Rio-Conference recommendations [15]. Since Rio [11], ESD approaches concentrating on sustainable, modified attitudes and behaviors have gradually been included from primary to higher education worldwide [15, 17]. To support learning, students need diverse access to educational contents, and therefore topics, skills and different teaching methods must vary [18]. Researchers have been interested in measuring ‘environmental awareness’, ‘attitudes based on connectedness to nature’ or ‘behavior towards the environment’ for several decades. An example of a standardized and world-wide accepted measuring instrument is the ‘Inclusion of Nature in Self (INS)’ scale [19], which was used in this study. By consulting another measuring technique, namely the ‘General Ecological Behavior (GEB)’ [20], five sub-scales where classified into sub-categories to describe the main category of ‘ecological behavior’. Another approach to ‘sustainable development’ claims that unprecedented material consumption, human greed and the human economic subsystem are huge problems of the modern world [21], which is why some authors see ESD critically. The concepts of ‘SD’ and ‘ESD’ are contradictory in their view of how ‘sustainability’ deals with the conflict between ‘economic growth’ and ‘environmental protection’ [22]. How far can natural resources [23] in developing countries be distributed fairly [24] and how is this problem related to ‘human welfare’, ‘equality’ and ‘equal rights’ [23]? Another point of discussion could be that people can choose between exploitation and protection of the environment [25] (*e*.*g*. by lifestyle or consumer behavior), subscribing to an anthrophocentrical or an ecocentrical view. Critics are concerned that sustainability tends towards anthropocentrism [23] if the rights and interests of human beings are the main focus. In contrast to that, the ecocentric approach puts special emphasis on the moral responsibility of humanity towards fellow humans [15], plants, animals and ecosystems [26]. Other authors argue that neither EE nor ESD solve crucial controversial disputes like ‘polyvalent decisions’. Replacing ‘nuclear power’ with ‘wind power systems’ brings new problems like ‘noise pollution’ and ‘bird protection’ [27]. It is particularly hard to raise students’ awareness of the value of nature [23] (*e*.*g*. ‘you will protect what you love but on the other hand, you will not protect what you don’t know’), because students have great difficulty understanding the underlying complex processes (*e*.*g*. why a forest dies) [27]. In addition, even ecologically oriented students struggle to deal with SD issues if they were taught by poorly trained teachers [23].

### Overview of Environmental Education history in school

The history of education suggests that EE can support children in achieving an eco-friendly way of life, not merely in acquiring knowledge about the bio-physical natural environment [28]. In the 60s, pupils gained only knowledge by studying species and physical systems. Later in the 70s practical knowledge was acquired through outdoor adventures and urban studies. The global education efforts of the 80s, which included for example [29] a variety of teaching methods (*e*.*g*. inquiry learning, problem-based learning, project based learning, case-based teaching, discovery learning or just in-time teaching) [18] already incorporated EE modules while ESD still was in its infancy. Since the 90s, EE has become a recognized approach around the globe and formal and informal efforts have been made to integrate cognitive, affective and psychomotoric aspects of learning [30]. However, diversity of teaching methods does not automatically lead to success, particularly if students’ have poor environmental knowledge, attitudes and behavior [31]. Not only ‘factual-knowledge’ but also ‘action-related knowledge’ and ‘effectiveness knowledge’ need to be increased [32] to promote positive environmental behavior. A few studies have examined short-term inputs (*e*.*g*. [33]) and residential program interventions [34], both of which have led to an increase of environmentally friendly attitudes and behavior. In our present study we monitor how freshmen perceive the terms EE and ESD after completing primary and secondary school during the UN decade. We assume that participants have some conceptions of EE and ESD, because their parents grew up during the evolutionary period of EE.

### Conceptions

Learning is an adaptive process where learners' conceptual schemes are progressively reconstructed by a wide range of experiences and ideas [35]. It is assumed that learners consider both naive personal and scientifically correct explanations [36]. Nowadays, students receive information from the media, which are not always scientifically based. Over a period of 25 years, Hansen [37] tested the knowledge of Norwegian students about environmental topics three times. He concluded that the students’ knowledge increased from the first to the last data collection. Furthermore, students were increasingly confused, perhaps because of the unlimited flood of information provided by *e*.*g*. media. Students often retain common sense beliefs and combine newly acquired school knowledge with their naive conceptions [38]. In addition to media, teachers also exert a significant influence on students’ conceptions. Çimer *et al*. [39] concluded that experienced teachers had more knowledge and fewer misconceptions than beginners. Teachers’ misconceptions should be eliminated before they are passed on to their students. Since the early 70s, conceptual ideas have been classified as pre-conceptions (*e*.*g*. [40]), misconceptions (*e*.*g*. [41, 42]), alternative conceptions (*e*.*g*. [43, 44]), common-sense concepts (*e*.*g*. [45]), initial conceptions (*e*.*g*. [46]) or individual perceptions (*e*.*g*. [47]). Conceptions of certain EE and ESD topics such as climate change (*e*.*g*. [48]), pollution (*e*.*g*. [49]), biodiversity (*e*.*g*. [50]) sustainability (*e*.*g*. [51]) or gene technology (*e*.*g*. [52]) have been studied in detail. To date there are no published studies about students’ conceptions of EE or ESD. Fröhlich and colleagues [53] concluded that the concepts of younger students concerning a specific topic differ from those of older students, because conceptions are age-dependent [54]. Pedagogical and curricular emphases vary in the different countries, and states and schools and have a fundamental influence on student’s conceptions [55].

### Research goals

It is our main goal to monitor those freshmen’s understanding of ‘EE’ and ‘ESD’ who grew up during the ‘UN Decade for Sustainable Development’. We had four objectives: first, to analyze conceptions of EE and ESD with the respect to three dimensions: ‘ecological’, ‘social’ and ‘economical’. Second, to detect the origins of individual environmental knowledge. Third, to analyze the relationship between humans and nature. Fourth, to determine the freshmen’s (unfulfilled) expectations of EE.

## Methods

### Ethics statement

The Ethics Committee of Northwest and Central Switzerland (EKNZ) has confirmed that the research project ‘How Freshmen perceive Environmental Education (EE) and Education for Sustainable Development (ESD)’ is in line with the general ethical and scientific standards for research with humans. It posed no health hazards in accordance with the Human Research Act (HRA, Article 51, paragraph 2). The project didn’t fall under the remit of the cantonal or federal law (Human research Act) and therefore an approval was not necessary by an ethic committee, because this project was not defined as a research project as per HRA Art. 2. All data privacy laws were respected. Gender, age and study status of participants were recorded pseudo-anonymously.

### Sample

Our sample consisted of 464 Swiss German freshmen from a variety of study programs (*e*.*g*., biology, pharmacy, economics; *N* = 464, *M* = 21.3, *SD* = *±* 3.1, male = 33.5%, female = 66.5%). All participants were confronted with three open and two closed questions testing individual concepts, knowledge and outlook concerning terms associated with the environment. The open questions included conceptions of Environmental Education (EE), Education for Sustainable Development (ESD) and individual (unfulfilled) expectations of EE. The first closed question listed six categories of EE sources (‘politics’, ‘advertisement’, ‘media’, ‘outreach’, ‘school’, ‘family’). All participants were asked to assess the individual importance of the sources of environmental knowledge using a four-point Likert-scale (‘1 = weak’, ‘2 = middling’, ‘3 = strong’ and ‘4 = very strong’). The second closed question was based on a 7-INS (Inclusion of Nature in Self; adapted from [19]) scale (scale: ‘A = very low’ to ‘G = very strong’) with two overlapping circles labelled ‘self’ and ‘nature’ to show the relationship to each other.

### Data analyses

**Fig 1:**
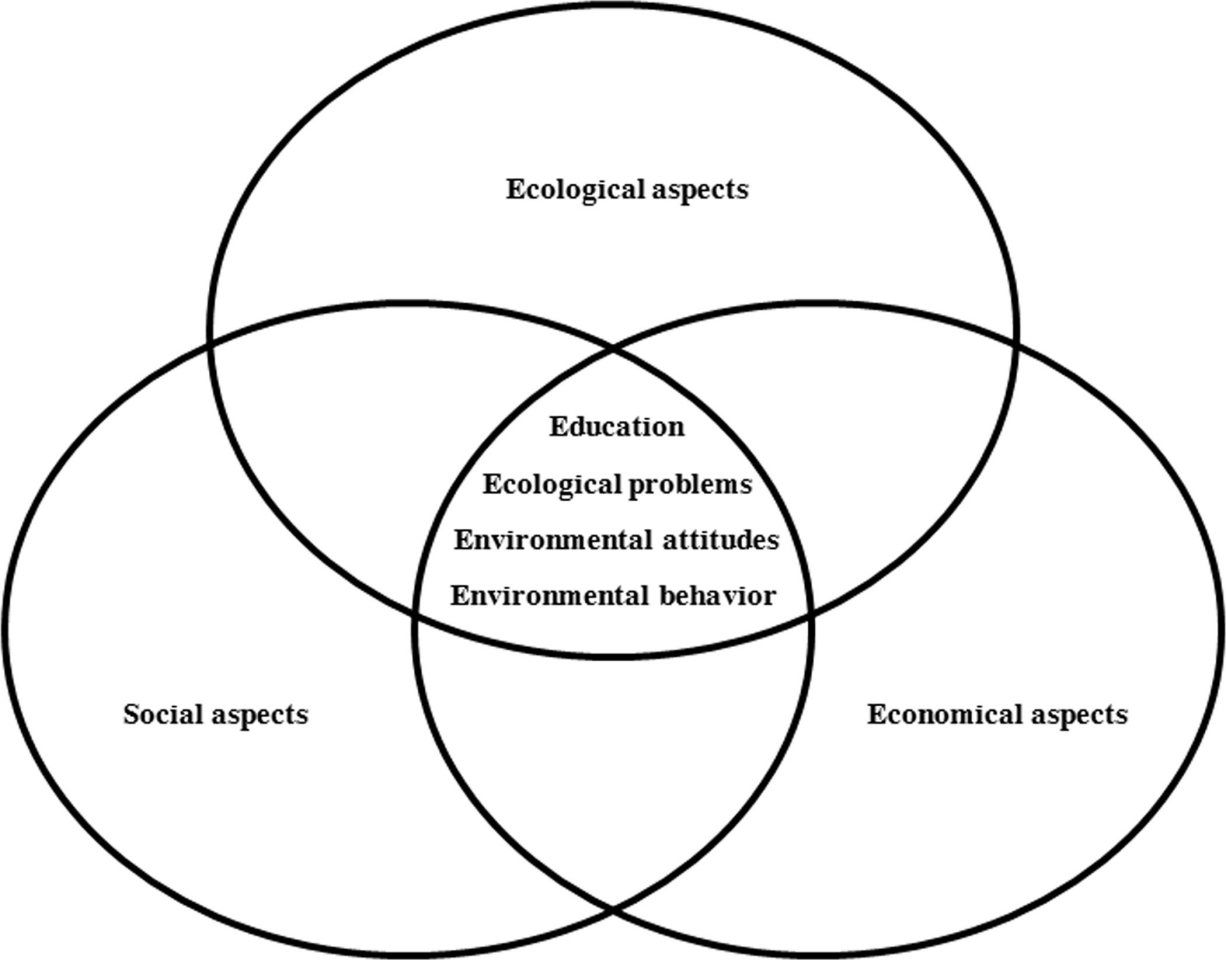
Categories of Environment Education and Education for Sustainable Development.

Statistical tests were conducted using R (Version 2.14.2). We analyzed both closed questions using Student’s T-Test, since the variables were normally distributed (Q-Q plot). Based on the current German syllabus [56], we extracted five main categories from the open questions inductively (ecological aspects, ecological problems, economical aspects, social aspects, education) and two main categories based on students’ statements deductively (environmental attitudes, environmental behavior) [57] (Fig 1).

### Categorization

To define the terms of ESD and EE more precisely, we used 28 identical sub-categories assigned to seven main categories (‘ecological aspects’, ‘economical aspects’, ‘social aspects’, ‘environmental behavior’, ‘environmental attitudes’, ‘ecological problems’ and ‘education’) (Table 1). In the cases of the question about EE (unfulfilled) expectations, we allocated 70 sub-categories. We summarized each main category as one vote, irrespective of the frequency with which each participant mentioned the sub-categories within each main category.

**Table 1:** Categorization examples from freshmen between EE and ESD.

		Main categories						
ID	Statements	1	2	3	4	5	6	7
**55**	**Recapturing(2) humans(4)** to the **environment(4)**, **becoming more conscious(2) and economical(6)** (EE).	0	1	0	1	0	1	0
	**Economically using resources(2)** and **preserving the environment(2)** (ESD).	0	1	0	0	0	0	0
**63**	**Information(1)**, built **awareness(2)** towards **nature(4)** and environment (EE).	1	1	0	1	0	0	0
	**Information(1)** about topics: **conservation of resources and handling(2): nutrition(6)**, **economy(3)**, **social(5)**, **environment(4)** (ESD).	1	1	1	1	1	1	0
**214**	**Promote ecological awareness(2)** (EE).	0	1	0	0	0	0	0
	**Learn(1)**, what we can do **to protect(2)** our **earth(4)** for **future generations(5)** (ESD).	1	1	0	1	1	0	0
**370**	**Learn(1)** how to **handle towards the environment(2)** (EE).	1	1	0	0	0	0	0
	To **teach(1) humans(4)**, that **resources(3) are limited** and **we should consume(6) only as much as we can produce(2)** (ESD).	1	1	1	1	0	1	0

Main categories: (1) Education, (2) Ecological attitudes, (3) Economical aspects, (4) Ecological aspects, (5) Social aspects, (6) Ecological behavior and (7) Environmental problems

To assign all participant statements to main or sub-categories, we accepted synonyms like nature or habitat instead of ‘Environment’ and information or lesson instead of ‘Education’ for ‘Environmental Education’. From 1443 observed statements we randomly selected 18% to assess the inter- and intra-reliability. We computed a score of 0.95 for inter-reliability and 0.86 for intra-reliability using Cohen’s Kappa Coefficient [58]. For the contingency analysis C_corr_ we set a limit of 0.2 and a significance level of α = 0.001. In our quantitative analysis we included the Bonferroni correction for both analyses separately.

## Results

All categories based on open questions (definitions, see Table 2), which are displayed exemplary on few examples in Table 1. A four-step analysis revealed the following: first, concept ideas about ESD and EE. Second, retrospectively labelled individual sources of EE. Third, the individual connectedness to nature in the Inclusion of Nature in Self (INS) and finally, (unfulfilled) expectations of EE issues in comparison to the freshmen’s individual concept ideas of EE.

**Table 2:** Defined categories of freshmen´s conceptions of Education for Sustainable Development (ESD) and Environmental Education (EE).

Category of conceptions	Definition	Examples
**Ecological aspects**	Interaction between organisms with other biotic and abiotic components of their environment.	organisms, nature, animals & plants, habitats
**Ecological problems**	Problems connected to environmental problems.	Environmental influence, pollution, climate change
**Social aspects**	The individual, in relation to its own social environment and thinking towards nature and fellow humans.	Sustainable lifestyle, next generation aspect
**Environmental attitudes**	Beliefs of people and society concerning nature, ecology and issues of the environment.	Awareness, connected with limited resources
**Economical aspects**	Economy resources and innovation.	Research, product/ resources, innovation
**Environmental behavior**	Behavioral patterns based on general ecological behavior (adjusted deductively from sub-scales of GEB) [20].	Consumption, waste avoidance, recycling
**Education**	Accumulation of individual knowledge.	Knowledge, information, understanding

**Fig 2:**
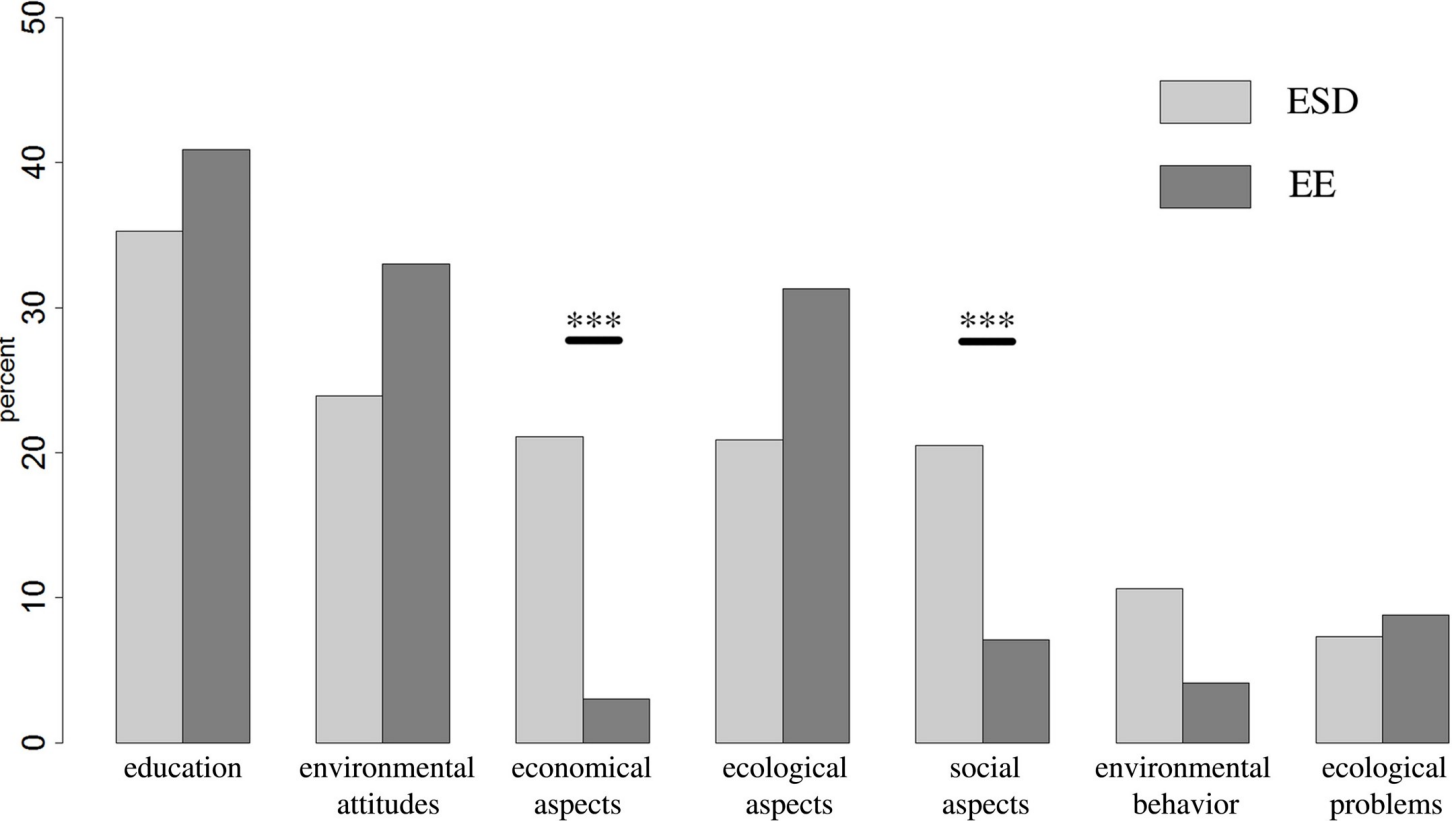
Percentage distribution of freshmen concept ideas of Education for Sustainable Development (ESD) and Environmental Education (EE). N_*participants*_ = 464.

A qualitative content analysis categorized students’ ideas about Environmental Education (EE) and Education for Sustainable Development (ESD) (Fig 2). We identified 1243 statements (n_ESD_ = 648, n_EE_ = 595) belonging to seven defined main categories (Table 1). A contingency analysis showed a relationship over all categories between ESD and EE (C_*corr*_ = 0.37, *n* = 1243, *p* < 0.001).

The main categories ‘social aspects’ (C_*corr*_ = 0.205, *n* = 1243, *p* < 0.001) and ‘economical aspects’ (C_*corr*_ = 0.296, *n* = 1243, *p* < 0.001) resulting this small effect (C_corr_ limit 0.2 and a significance level of 0.001, see method).

**Fig 3:**
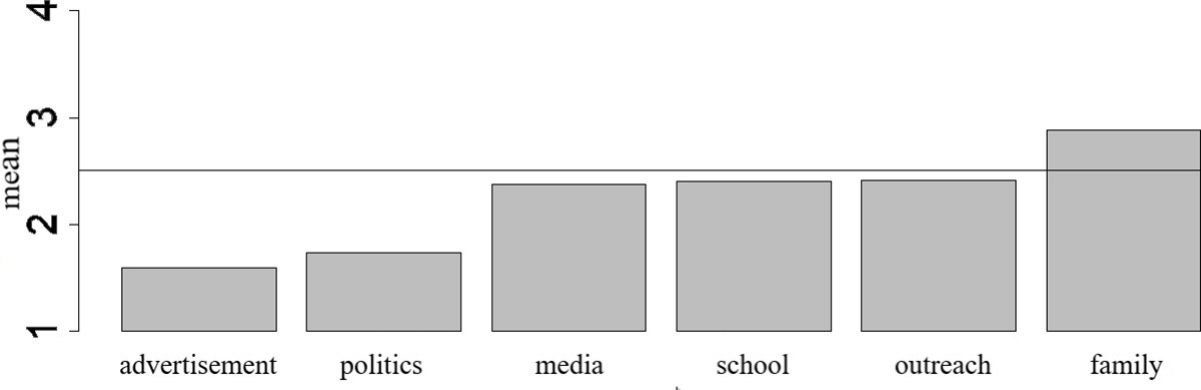
Comparison of overall mean scores when retrospectively labelling individual sources of Environmental Education (*N* = 464).

**Fig 4:**
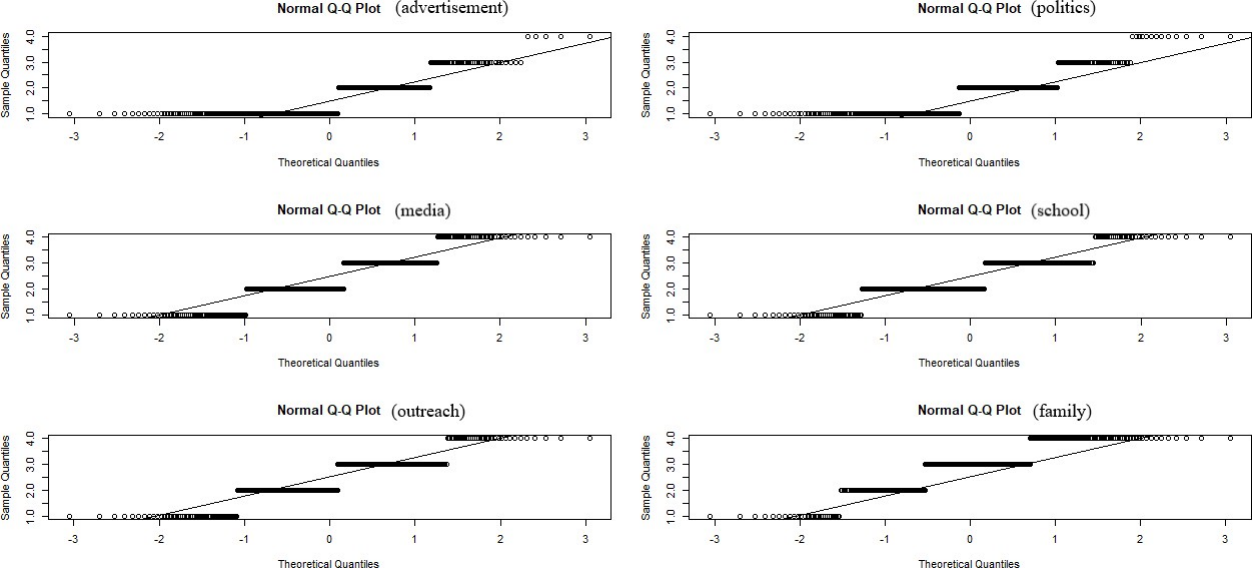
All Q-Q plot graphics of EE sources show a normally distributed data based on the Likert-scale.

A quantitative analysis yielded six categories of sources of Environmental Education: ‘advertisement’ (*M* = 1.592, *SD* = 0.727), ‘politics’ (*M* = 1.733, *SD* = 0.786), ‘media’ (*M* = 2.377, *SD* = 0.879), ‘school’ (*M* = 2.406, *SD* = 0.768), ‘outreach’ (*M* = 2.411, *SD* = 0.830) and ‘family’ (*M* = 2.880, *SD* = 0.846) (Fig 3). A Paired Student’s T-Test after testing normal distribution (Fig 4) indicated differences between the categories ‘advertisement’ and ‘politics’ (*t* = -3.251, *df* = 436, *p* = 0.001), ‘advertisement’ and ‘media’ (*t* = -17.197, *df* = 435, *p* < 0.001), ‘advertisement’ and ‘school’ (*t* = -16.062, *df* = 433, *p* < 0.001), ‘advertisement’ and ‘outreach’ (*t* = -15.817, *df* = 430, *p* < 0.001), ‘advertisement’ and ‘family’ (*t* = -24.686, *df* = 439, *p* < 0.001), ‘politics’ and ‘media’ (*t* = -12.192, *df* = 435, *p* < 0.001), ‘politics’ and ‘school’ (*t* = -13.076, *df* = 433, *p* < 0.001), ‘politics’ and ‘outreach’ (*t* = -12.524, *df* = 430, *p* < 0.001), ‘politics’ and ‘family’ (*t* = -22.908, *df* = 438, *p* < 0.001), ‘media’ and ‘family’ (*t* = -9.188, *df* = 436, *p* < 0.001), ‘school’ and ‘family’ (*t* = -8.740, *df* = 434, *p* < 0.001) and the categories ‘outreach’ and ‘family’ (*t* = -8.807, *df* = 431, *p* < 0.001). The effect size of all analysis explain less than 8% variance between two category pairs.

**Fig 5:**
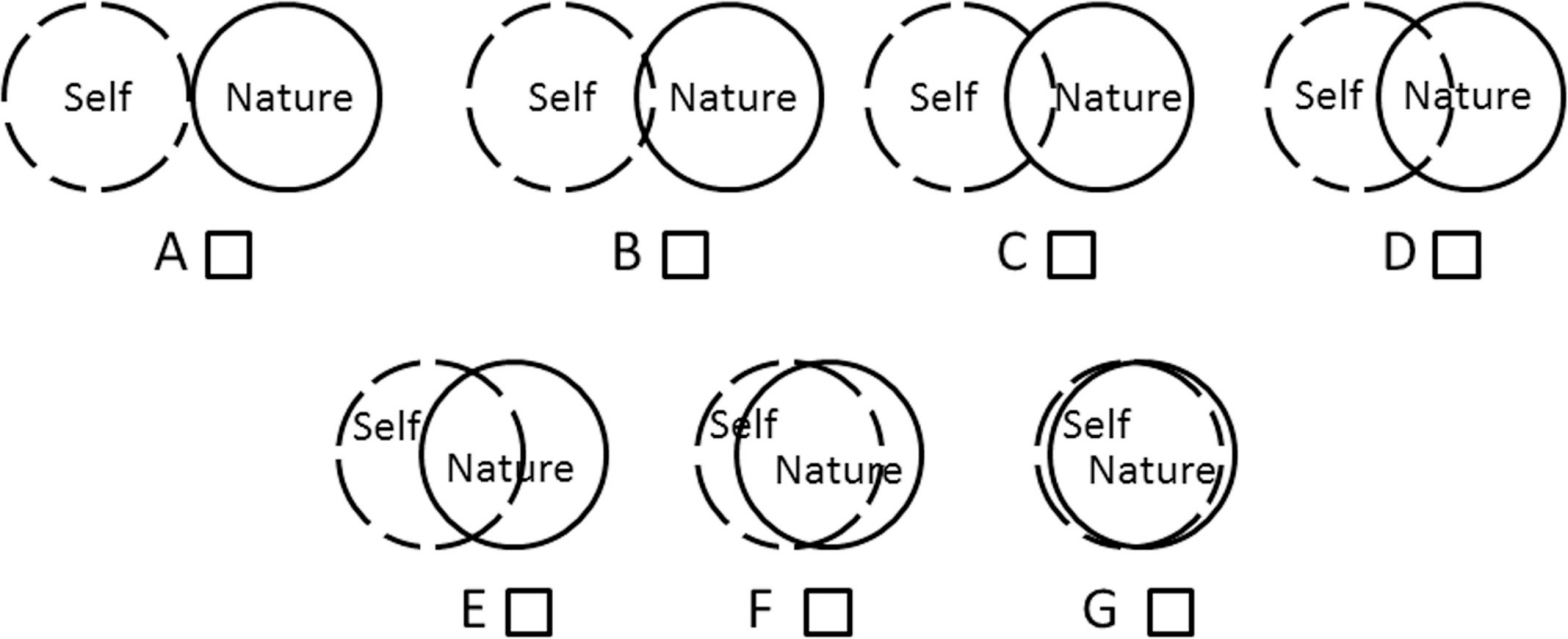
INS adapted with two overlapping circles labelled ‘nature’ and ‘self’ [19].

The Inclusion of Nature in Self scale (INS) [19] describes the relationship between nature and the self (Fig 5).

**Fig 6:**
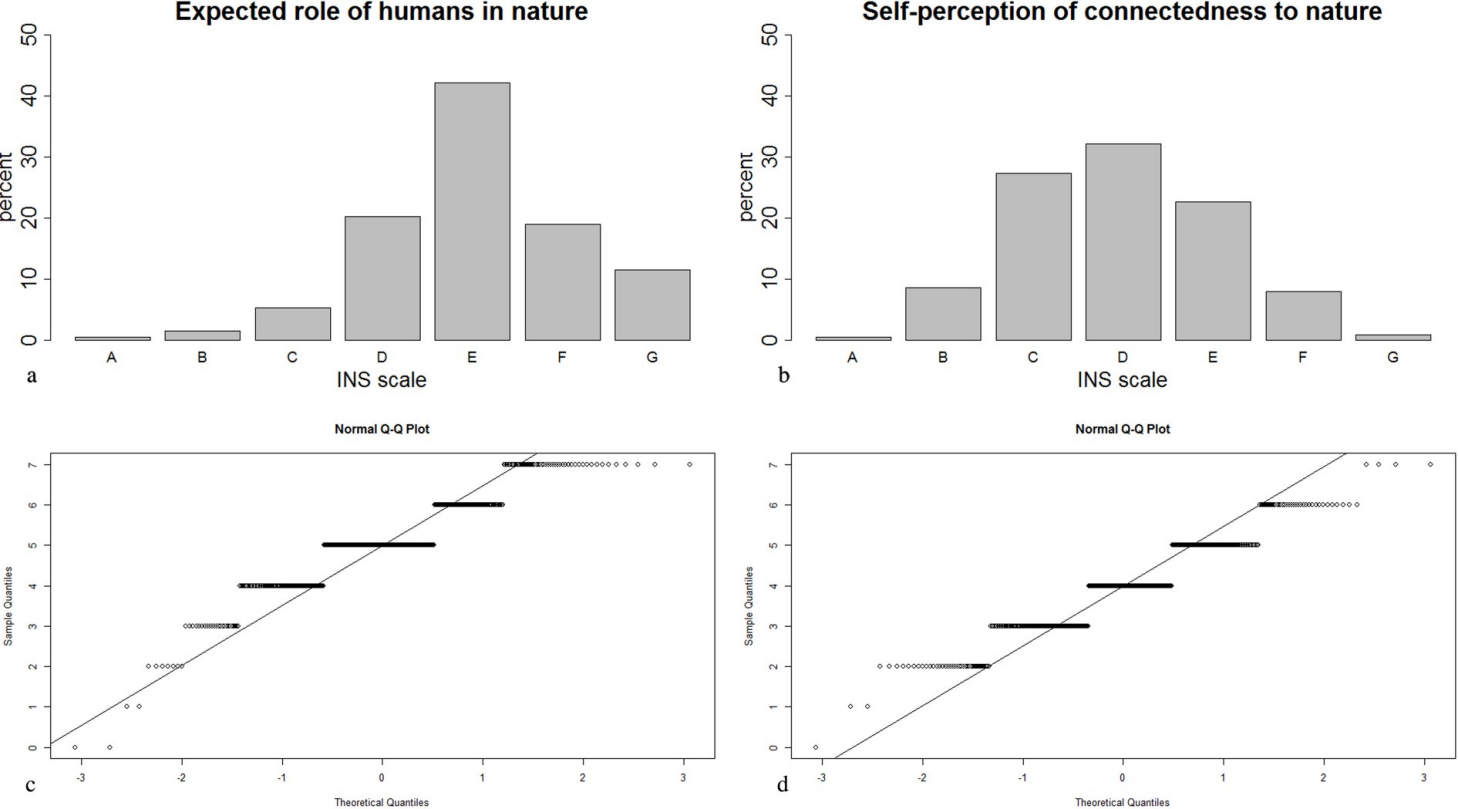
Connectedness to nature: ‘human-perception’ (a, c) and ‘self-perception’ (b, d) including Q-Q plot graphics showing normally distributed data based on the Likert scale.

A Paired Student’s T-Test indicated a difference between ‘self-perception’ (*M* = 3.954, *SD* = 1.145) and ‘human-perception’ (*M* = 5.024, *SD* = 1.174) (Fig 6A and 6B) with respect to connectedness to nature (*t* = 20.5, *df* = 451, *p* < 0.001). A moderate effect (*r* = 0.48) explains 23.04% of the dependency between them.

**Fig 7:**
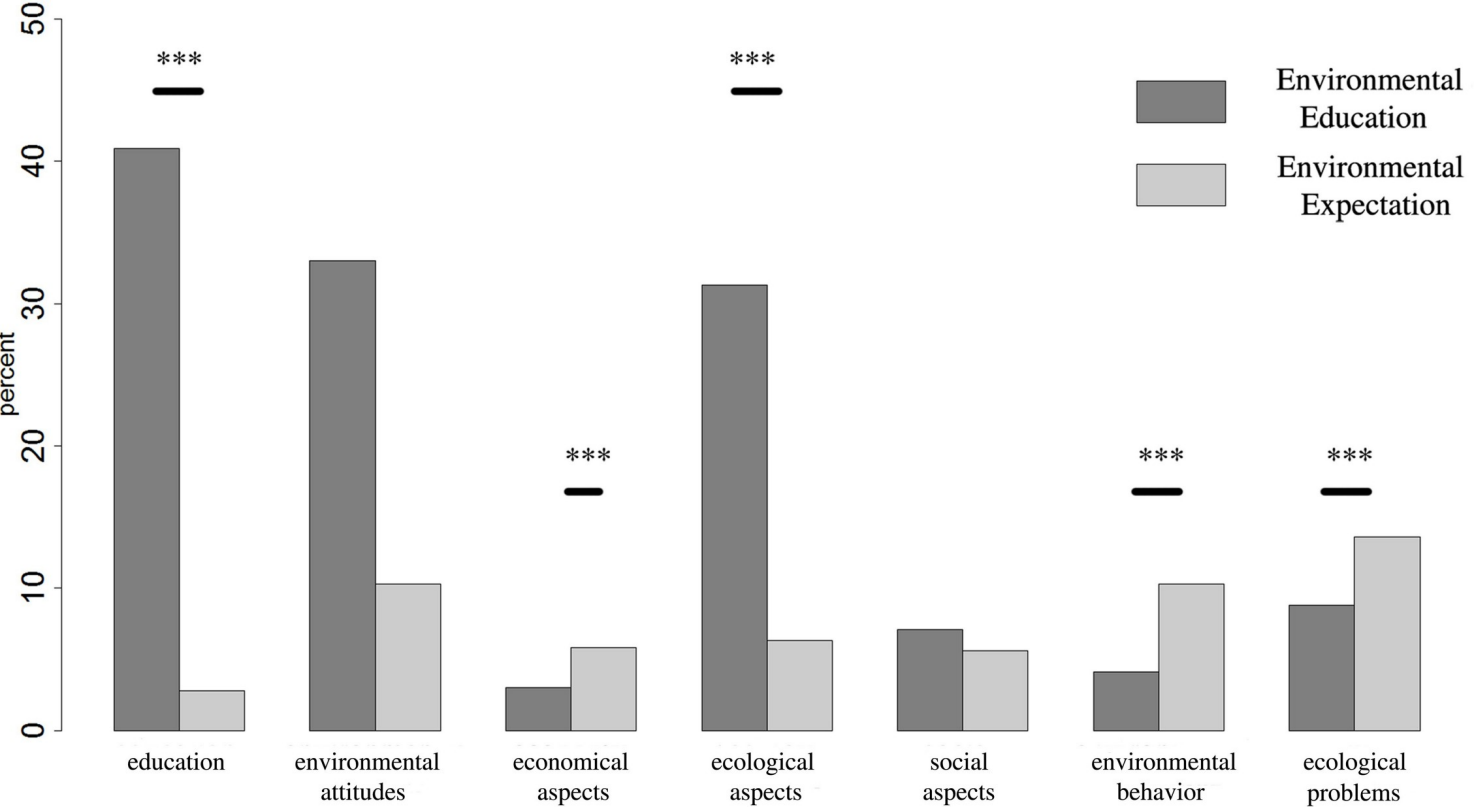
Conceptions about ‘Environmental Education (EE)’ (row of order see Fig 2) and individual ‘environmental expectations’.

The second qualitative content analysis categorized students’ ideas about ‘Environmental Education’ (Fig 2) and ‘(unfulfilled) Environmental Expectation’ (Fig 7). We identified 849 statements (n_*Environmental*Education_ = 595, n_(unfulfilled)*EnvironmentalExpectation*_ = 254) belonging to seven defined main categories (Table 2). A contingency analysis showed a relationship over all categories between ‘Environmental Education’ and ‘(unfulfilled) Environmental Expectation’ (C_*corr*_ = 0.536, *n* = 849, *p* < 0.001).

The main categories ‘education’ (C_*corr*_ = 0.385, n_*observed*_ = 203, n_*not observed*_ = 646, *p* < 0.001) and ‘ecological aspects’ (C_*corr*_ = 0.201, n_*observed*_ = 174, n_*not observed*_ = 675, *p* < 0.001) produce a significantly higher number of ‘Environmental Education’ statements in comparison to fewer (unfulfilled) ‘Environmental Expectation’ statements. On the other hand, we obtained fewer statements in the main categories ‘ecological problems’ (C_*corr*_ = 0.334, n_*observed*_ = 104, *n*_*not observed*_ = 745, *p* < 0.001), ‘environmental behavior’ (C_*corr*_ = 0.357, n_*observed*_ = 67, n_*not observed*_ = 782, *p* < 0.001) and ‘economical aspect’ (C_*corr*_ = 0.237, n_*observed*_ = 41, n_*not observed*_ = 808, *p* < 0.001) if compared with a higher ‘(unfulfilled) Environmental Expectation’ based on our definition (C_corr_ limit 0.2 and a significant level of 0.001, see method).

## Discussion

### The ‘ecological’ dimension in ESD and EE

In 2015, 17 Sustainable Development Goals (SDGs) were formulated, including the basic ecology of local and global ecosystems (*e*.*g*. 13 ‘climate change’, 14 ‘life below water’ or 15 ‘life on land’) [59]. The roots of the definition of ‘ecology’–linking the biotic and abiotic world–go back to Aristotle, Buffon, Wallace, Darwin or Haeckel [60]. For one in three participants, we observed a minimum of one statement in the main category of ‘ecological aspects’ in EE, but only for one in five in ESD. EE clearly contained more statements in the sub-categories of ‘habitat’ and ‘ecosystem/environmental impacts’ than did ESD. In both EE and ESD, we observed few statements concerning ‘animals’, ‘plants’ or ‘humans’. Within the ‘ecological’ category, we combined statements about ‘climate change’, ‘environment pollution’ and ‘environment influences’ as sub-categories of the main category ‘ecological problems’. Less than 10% of participants mentioned one statement in EE, or in both EE and ESD, although *e*.*g*. ‘climate change’ is one of the essential focuses in ESD [61] and–next to ‘micro plastic’ [62], ‘hormones in rivers and lakes’ [63] or ‘sunscreen particle’ in oceans [64]–the greatest threat to our environment [65, 66].

### The aspect of the ‘social’ dimension in ESD and EE

The ‘social’ category–as an essential environmental issue–has commonly been recognized as the weakest ‘pillar’ of sustainable development [13, 67]. We assigned for one in five participants a minimum of one statement to the main category of ‘social aspects’ in ESD, and for one in fourteen participants in EE. The effect size in the category ‘social’ is small, but was perceived from freshmen stronger in ESD than in EE. This is quite in line with a recent study with over 2400 Swedish students, where ESD was assigned an essential role in a more sustainable future [68]. Further, the ‘next generation aspect’, where we observed 23 statements, might provide a first indication of more thoughtful behavior towards value-oriented decisions [27]. In a Brazilian study 68% of all participants were confused when they were asked questions about their opinion of ESD, because in their past they were familiar only contact with EE. EE already contains ‘social’ and ‘economical’ elements [69]. Other social issues like ‘employment’, ‘human rights’, ‘gender equity’, ‘peace’ or ‘human security’, although regarded as essential [63], appeared neither in EE nor ESD in our sample.

### The aspect of the ‘economical’ dimension in ESD and EE

Economic growth with all its effects on society and environment is expected to be a key concept of ESD [69]. However, in our case one in five participants yielded a minimum of one statement in the main category of ‘economical aspects’ in ESD. In contrast, only one in 34 participants did so for EE. Similar to Manni and colleagues [70], we counted the word resources in the various main categories 165 times in ESD and 65 times in EE. In our opinion, it makes a difference whether the concept of ESD contains resources (as a single word, impersonal) or ‘conservation of resources and handling’ (personal). For example, the statement of one freshman: ‘sparingly using resources and preserving the environment’ fits best to the main category ‘environmental attitudes’ (Table 1) following our definition (Table 2). Unfortunately, we cannot tell if this statement was made in reference to a social (*e*.*g*. next generation aspect), economical (sparingly consume to save money) or ecological background (avoid products with palm oil to protect rain forests). Within the main category of ‘environmental attitudes’, we observed 70 statements in the EE subcategory of ‘appreciate/perceive/preserve the environment’ and only 18 statements in the same sub-category of ESD. The same applies to all the other sub-categories within the main category of ‘environmental attitudes’ (‘awareness of/responsibility for nature’, ‘to save resources’ and ‘environmental protection’). This shows a countertrend to the definition of environment [2] as found in ESD. In conclusion, the conceptual patterns clearly do not follow the protocol of ‘economical’, ‘ecological’ and ‘social’ aspects as single dimensions of EE and ESD. On average, each participant mentioned statements of only two out of seven main categories. Additionally, the results of the contingency analysis revealed the frequency between the categories and their classification to EE and ESD based on students’ perception. In conclusion we had a small effect size over all categories, which was derivate from the categories of ‘social’ and ‘economical aspects’.

### Environmental Education in relation to lifetime learning aspect and connectedness to nature

Although the frequencies of conceptions differed, the most important source for EE is ‘family'. Other studies have reported this for earlier age-groups: Eagles & Demare [71] reported for 6^th^-graders that talking about the environment at home while watching nature films, and reading about the environment were the most frequent sources of EE. ‘Family‘ is apparently also important for the age group in our study and seems to be an imprinting factor on individuals’ attitudes towards and knowledge about EE. Pe’er and colleagues [72] described a significant positive relationship between the mother’s education (as an indicator of socioeconomic status) and a student’s environmental knowledge and attitudes. They assume that growing up in a well-educated family supports more pro-environmental attitudes. Further, they found out that well educated individuals had greater exposure to ecological ideas than less educated individuals. EE and/or ESD may need a long period–from early childhood throughout adolescence to adulthood–to become established. In our sample, it is not clear which type of media contributes to most EE conceptions. Even eLearning tools such as HOBOS, is an outstanding means of replacing direct experience of nature by observing beehives remotely [73]. Commercial advertising (*e*.*g*. flyers and posters) and politics seem to play a very minor role in EE in our sample. In conclusion, long-term sources such as family or school, including different kinds of media (*e*.*g*., TV, journals and books), are perceived as the most important sources in EE (Fig 3). Connectedness to nature, as a common goal for ‘Environmental Education Programs’ in schools [74], is expected to positively influence individual environmental behavior (*e*.*g*. [32]). Our results (that younger students are more engaged than older ones) are in accordance with the literature (*e*.*g*. [75]). In addition, differences appeared between an anthropocentric self-perception view based on the Inclusion of Nature in Self (INS) scale (Fig 6A) and the overall view of the relationship between humans and nature (Fig 6B).

### Environmental Education (unfilled) expectations

Less than half of our participants replied to the open question concerning individual expectations of EE, although low scores were observed over all categories in general. Frequent categories like ‘ecological aspects’, ‘environment attitudes’ and ‘education’ were infrequent, while other main categories such as ‘environmental behavior’, ‘economical aspects’ and ‘ecological problems’ were observed more frequently. The frequent questions of freshmen about topics like: ‘how to protect the environment’, ‘how to avoid waste’ or ‘how to encouraged learning ecologically sensitive behavior’ demonstrate, that the required environmental knowledge has been conveyed insufficiently or not even at all at school for example. Nevertheless, individual statements in relation to anthropocentric impacts such as ‘climate change’, ‘global warming’, ‘carbon dioxide emissions’ and other ‘harmful environmental influences’ in the main category of ‘ecological problems’ occurred less frequently than expected, although topics like ‘climate change’ (*e*.*g*. [61]), ‘micro plastic’ (*e*.*g*. [62]) or ‘hormones in rivers and lakes’ (*e*.*g*. [63]) have a strong media presence. The most frequent observation in the main category of ‘ecological behavior’ occurred in the sub-categories of nutrition consumptions (*e*.*g*. regional/seasonal, alternative or genetically engineered foods). In the main category ‘economical aspects’ terms like innovation and alternative energies’ were mentioned often. Additionally, the results of the contingency analysis revealed the frequency between the categories and their classification to EE and EE Expectation, based on students’ perception. In conclusion we had a small effect size over all categories, which was derivate from the categories of ‘education’, ‘economical aspects’, ‘ecological aspects’, environmental behavior’ and ‘ecological problems’.

## Conclusion

The sustainable aspect according to the Rio-conference [11] is in line with a newly observed sub-category named ‘next generation’ and only included in ESD, which is considered as an expansion of EE. Higher numbers of statements in the sub-categories of ‘avoiding waste’ and ‘alternative consumptions’ (*e*.*g*. regional/seasonal, alternative or genetically engineered foods) arise from the category ‘environmental behavior’ in a clear development towards sustainability in ESD. The term resources was observed more frequently in ESD, 165 times in contrast to 65 times in EE. It is pleasing that freshmen obviously wanted more information on topics like ‘renewable energies’ or ‘innovations’ in the category of ‘economical aspects’, presented as an open question in (unfulfilled) EE expectations. This may show a general tendency towards economic growth, although this topic was not included in questions about (unfulfilled) expectations in ESD. Derived from this example and others, we assume that the perceptions of freshmen are composed of two coexisting approaches with overlapping conceptions in EE and ESD. Based on the freshmen’s strong limited ecological conceptions about habitats and humans, we counted fewer observations of these terms in ESD than in EE. In summary: fewer concepts in the category ‘environmental attitudes’, may not be in line with the original definition of environment in EE [2] and suggest a trend away from the ecocentric view. This observation was confirmed by the results of the Inclusion of Nature in Self (INS) scale of connectedness to nature, which showed that freshmen think of themselves as quite anthropocentric and yet are simultaneously convinced that an ecocentric world view is the ideal (Fig 6A and 6B). Although general interest in ‘(unfulfilled) environmental expectation’ was low, the highest rate was observed in the main category of ‘environmental problems’ including current ecological problems like climate change. Retrospectively, family, school (especially teachers), outreach and media seem to be the most important sources of EE in our sample: they are crucial points of contact from early childhood to adulthood and help young people to become responsible citizens.

## Supporting information

S1 Dataset Dataset of EE and ESD conceptions. (XLSX)

## References

[1] Rosaleen J. Outdoor learning: past and present: Past and Present. Berkshire, England: Open University Press; 2012. 144 p.

[2] IUCN. International Working Meeting on Environmental Education in the School Curriculum, Final Report. Gland, Switzerland: IUCN; 1970.

[3] UNESCO. The Stockholm Declaration. Stockholm: UNESCO 1972.

[4] Carson R. Silent Spring Boston, MA: Houghton Mifflin Co.; 1962. 363 p.

[5] George JL, Frear DEH. Pesticides in the Antarctic. J Appl Ecol. 1966; 3:155–67.

[6] McCormick J. The global threat of acid pollution. London; 2009. 257 p.

[7] Seinfeld JH, Pandis SN. Atmospheric Chemistry and Physics: From Air Pollution to Climate Change. New York: John Wiley & Sons; 1998. 1191 p.

[8] Parson E. Protecting the Ozone Layer: Science and Strategy New York: Oxford University Press; 2003.

[9] UNESCO-UNEP. The Belgrade Charter: A global framework for environmental education. Connect: UNESCO-UNEP Environmental Education Newsletter; 1976, 1(1): 1–2.

[10] UNESCO UNEP. The Tbilisi Declaration: Final report intergovernmental Conference on Environmental Education. Paris, France: UNESCO ED/MD/49; 1978. p. 1–96.

[11] UN. Rio Declaration on Environment and Development—Preamble. 1992.

[12] vonCarlowitz HC. Sylvicultura oeconomica oder hauswirtschliche Nachricht und naturmassige Anweisung zur wilden Baumzucht nebst grundlicher Darstellung/Wie zu fordest durch Gottliche Benehmen dem allenthalben und insgemein eintreffenden Grossen Holz/Mangel. Erben, Leipzig; 1732.

[13] Lehtonen M. The environmental-social interface of sustainable development: Capabilities, social capital, institutions. Ecol Econ. 2004; 49(2):199–214.

[14] Reilly S, Petrillo DH, Demchik M. Environmental Education’s Role in Sustainable Development: Three Case Studies from India, South Africa & the United States. Int Resour Manag. 2008; (NR 523).

[15] UNESCO. The UN Decade for Education for Sustainable Development (DESD 2005–2014): the first two years. Paris; 2007.

[16] McKeown R. Education for sustainable development toolkit. Version 2. Knoxville, Tennessee; 2002.

[17] Gibson HL, Chase C. Longitudinal Impact of an Inquiry-Based Science Program on Middle School Students’ Attitudes Toward Science. Sci Educ. 2002; 86(5):693–705. 10.1002/sce.10039.

[18] Prince M, Felder R. Inductive teaching and learning methods: definitions, comparisons, and research bases. J Eng Educ [Internet]. 2006; 95(2):123–38. Available from: http://onlinelibrary.wiley.com/doi/10.1002/j.2168-9830.2006.tb00884.x/abstract.

[19] Schultz. Inclusion with nature: Understanding the psychology of human–nature interactions In Schmuck P & W P., (Eds.), Development T psychology of sustainable, (pp. 61–78). New York: Kluwer.

[20] Kaiser FG, Oerke B, Bogner FX. Behavior-based environmental attitude: Development of an instrument for adolescents. J Environ Psychol. 2007; 27(3):242–51.

[21] Rees WE. More jobs, less damage: a framework for sustainability. Altern J. 1995; 21(4):24–30.

[22] Czech B. Prospects for reconciling the conflict between economic growth and biodiversity conservation with technological progress. Conserv Biol. 2008; 22(6):1389–98. 10.1111/j.1523-1739.2008.01089.x 19076872

[23] Kopnina H. Education for sustainable development (ESD): The turn away from ‘environment’ in environmental education? Environ Educ Res. 2012; 18(5):699–717.

[24] Banerjee SB. Who Sustain Whose Development? Sustainable Development and the Reinvention of Nature. Organ Stud [Internet]. 2003; 24(1):143–80. 77.

[25] McCormick J. Reclaiming Paradise: The Global Environmental Movement Vol 660 Indiana University Press, Bloomington; 1991. 263 p.

[26] Kopnina H. Revisiting education for sustainable development (ESD): Examining anthropocentric bias through the transition of environmental education to ESD. Sustain Dev. 2014; 22:73–83.

[27] Rost J. Umweltbildung—Bildung für nachhaltige Entwicklung. Was macht den Unterschied? [Environmental Education–Education for Sustainable Development. What is the difference between them?]. Zeitschrift für Internationale Bildungsforschung und Entwicklungspädagogik)[Journal International Eduction research development pedagogy]. 2002; 25:7–12.

[28] UNESCO-IEEP. Environmental Education: Module for Pre-Service Training of Science Teachers and Supervisors for Secondary Schools. In: Environmental Educational Series. 1985. p. 123.

[29] Palmer JA. Environmental Environmental Education In The 21ST Century Theory, practice, progress and promise. London (Routledge); 1998.

[30] De Kock A, Sleegers P, Voeten MJM. New Learning and the Classification of Learning. Rev Educ Res. 2004; 74(2):141–70. 10.3102/00346543074002141.

[31] Rickinson M. Learners and Learning in Environmental Education: A Critical Review of the Evidence. Environ Educ. 2001; 7(3):207–317. 10.1080/13504620120065230.

[32] Kaiser FG, Roczen N, Bogner FX. Competence Formation in Environmental Education: Advancing Ecology-Specific Rather Than General Abilities. Umweltpsychologie [Environmental Psychology]. 2008; 12(2):56–70.

[33] Fremerey C, Bogner F. Learning about Drinking Water: How Important are the Three Dimensions of Knowledge that Can Change Individual Behavior? Educ Sci [Internet]. 2014; 4:213–228. 10.3390/educsci4040213.

[34] Liefländer AK, Bogner FX. The effects of children’s age and sex on acquiring pro-environmental attitudes through environmental education. J Environ Educ. 2014; 45(2):105–117. 10.1080/00958964.2013.875511.

[35] Driver R. Students’ conceptions and the learning of science. Int J Sci Educ. 1989; 11(5):481–490.

[36] Andresen L, Boud D, Cohen R. Experience-based learning In: Foley G (Ed), Understanding adult education and training (2nd ed, pp 225–239) [Internet]. Sydney: Allen & Unwin.; 1999 Available from: http://complexworld.pbworks.com/f/Experience-basedlearning.pdf.

[37] Hansen PJK. Knowledge about the Greenhouse Effect and the Effects of the Ozone Layer among Norwegian Pupils Finishing Compulsory Education in 1989, 1993, and 2005-What now? Int J Sci Educ. 2010; 32(3):397–419. 10.1080/09500690802600787.

[38] Floden RE, Buchmann M. 'Breaking with Everyday Experience for Guided Adventures in Learning', in Buchmann M and Floden M (eds). Detachment and Concern: Conversations the Philosophy of Teaching and Teacher Education, Cassell, London.

[39] Çimer OS, Çimer A, Ursavas N. Student teachers’ conceptions about global warming and changes in their conceptions during pre-service education: a cross sectional study. Educ Res Rev. 2011; 6(8):592–7.

[40] Novak JD. A Theory of Education. NY, USA: Cornell University Press; 1977.

[41] Doran RL. Misconception of selected science concepts held by elementary school students. J Res Sci Teach. 1972; 9:127–37. https://doi.org/doi:10.1002/tea.3660090204.

[42] Schmid S, Bogner FX. Is there more than the sewage plant? University freshmen’s conceptions of the urban water cycle. PLoS One. 2018 13(7): 1–14. 10.1371/journal.pone.0200928. PMC6053198 30024937

[43] Driver R, Easley J. Pupils and Paradigms: a Review of Literature Related to Concept Development in Adolescent Science Students. Stud Sci Educ. 1978; 5(1):61–84. 10.1080/03057267808559857.

[44] Thorn CJ, Bissinger K, Thorn S, Bogner FX. Trees live on soil and sunshine!—Coexistence of scientific and alternative conception of tree assimilation. PLoS One [Internet]. 2016; 11(1). Available from: 10.1371/journal.pone.0147802. PMC4725716 26807974

[45] Halloun IA, Hestenes D. The initial knowledge state of college physics students. Am J Phys. 1985; 53:1043–1055.

[46] Chi MTH, Slotta JD, Leeuw N De. From things to processes: a theory of conceptual change for learning science concepts. Learn Instr. 1994; 4:27–43.

[47] Schönfelder ML, Bogner FX. Individual perception of bees: Between perceived danger and willingness to protect. PLoS One. 2017; 12(6):1–16. 10.1371/journal.pone.0180168. PMC5491143 28662124

[48] Sellmann D, Bogner FX. Climate Change and the Sustainable Use of Water Resources—Chapter 47: Educational in Global Climate Change at a Botanical Garden: Student’s Perceptions and Inquiry-Based Learning Walter Leal Filho, editor. Berlin: Springer-Verlag; 2012. 779–786 p.

[49] Brody MJ. Understanding of Pollution among 4th, 8th, and 11th Grade Students. J Environ Educ. 1991; 22(2). 10.1080/00958964.1991.9943051.

[50] Kilinc A, Yeşiltaş NK, Kartal T, Demiral Ü, Eroǧlu B. School Students’ Conceptions about Biodiversity Loss: Definitions, Reasons, Results and Solutions. Res Sci Educ. 2013; 43(6):2277–307. 10.1007/s11165-013-9355-0.

[51] Walshe N. Understanding students’ conceptions of sustainability. Environ Educ Res. 2008; 14(5):537–58. 10.1080/13504620802345958.

[52] Franke G, Scharfenberg F, Bogner FX. Investigation of Students’ Alternative Conceptions of Terms and Processes of Gene Technology. ISRN Educ. 2013;2013 10.1155/2013/741807.

[53] Fröhlich G, Goldschmidt M, Bogner FX. The effect of age on students’ conceptions of agriculture. Stud Agric Econ [Internet]. 2013; 115(1):61–67. Available from: 10.7896/j.1301.

[54] Trowbridge JE, Mintzes JJ. Alternative conceptions in animal classification: A cross-age study. J Res Sci Teach. 1988; 25(7):547–71. 10.1002/tea.3660250704.

[55] Lederman N. Students’ and teachers’ conceptions of the nature of science: A review of the research. J Res Sci Teach [Internet]. 1992; 29(4):331–359. Available from: http://onlinelibrary.wiley.com/doi/10.1002/tea.3660290404/abstract.

[56] KMK. (2003). Richtlinien für die Umweltbildung an den bayerischen Schulen. [Guidelines for Bavarian schools in environmental education]. ISB, München [Munich]; 2003.

[57] Mayring P. Qualitative Content Analysis. Qual Soc Res. 2000; 1(2).

[58] Cohen J. A coefficient for agreement for nominal scales. Educ Psychol Meas. 1960; 20:37–46.

[59] UN. The Sustainable Development Goals Report. 2017.

[60] Mc.Comas WF. The ideal environmental science curriculum: I. History, rationale, misconceptions & standards. Am Biol Teach. 2002; 64(9):665–72. 10.1662/0002-7685(2002)064[0665:TIESCI]2.0.CO;2.

[61] Crate SM, Nuttall M (Eds.). Anthropology and Climate Change: From Encounters to Action. Left Coast Press, Walnut Creek, CA; 2007.

[62] Ivar do Sul JA, Costa MF. The present and future of microplastic pollution in the marine environment. Environ Pollut. 2014; 185:352–64. 10.1016/j.envpol.2013.10.036 24275078

[63] Kolpin DW, Furlong ET, Meyer MT, Thurman EM, Zaugg SD, Barber LB, et al Pharmaceuticals, hormones, and other organic wastewater contaminants in U.S. streams, 1999–2000: A national reconnaissance. Environ Sci Technol. 2002; 36(6):1202–11. 11944670 10.1021/es011055j

[64] Downs CA, Kramarsky-Winter E, Segal R, Fauth J, Knutson S, Bronstein O, et al Toxicopathological Effects of the Sunscreen UV Filter, Oxybenzone (Benzophenone-3), on Coral Planulae and Cultured Primary Cells and Its Environmental Contamination in Hawaii and the U.S. Virgin Islands. Arch Environ Contam Toxicol. 2016; 70(2):265–88. 10.1007/s00244-015-0227-7. 26487337

[65] Fischlin A, Midgley GF, Price JT, Leemans R, Gopal B, Turley C, et al. Ecosystems, their properties, goods and services. Climate Change 2007: Impacts, Adaptation and Vulnerability. Contribution of Working Group II to the Fourth Assessment Report of the Intergovernmental Panel on Climate Change (Chapter 4), UK: Cambridge University Press; 2007. 211–272 p.

[66] UNESCO. United Nations Decade of Education for Sustainable Development (2005–2014): International Implementation Scheme. Paris; 2005.

[67] Woolcock M. The Place of Social Capital in Understanding Social and Economic Outcomes. In John F. Helliwelled. The Contribution of Human and Social Capital to Sustained Economic Growth and Well-Being (Ottawa: HDRC)(Proceedings of an OECD/HRDC conference, Quebec, March 19–21, 2000).

[68] DeBoeve JP, Gericke N, Olsson D, Berglund T. The Effectiveness of Education for Sustainable Development. Educ Sustain Dev Sustain. 2015; 7(11). 10.3390/su71115693.

[69] Gadotti M. Education for Sustainability: A Critical Contribution to the Decade of Education for Sustainable Development. Green Theory Prax J Ecopedagogy. 2008; 4(1):15–64.

[70] Manni A, Sporre K, Ottander C. Mapping What Young Students Understand and Value Regarding Sustainable development. Int Electron J Environ Educ. 2013; 3(1):17–35.

[71] Eagles PFJ, Demare R. Factors Influencing Children’s Environmental Attitudes. J Environ Educ. 1999; 30(4):33–7.

[72] Pe’er S, Goldman D, Yavetz B. Environmental Literacy in Teacher Training: Attitudes, Knowledge, and Environmental Behavior of Beginning Students. J Environ Educ [Internet]. 2007;39(1):45–59. Available from: http://www.tandfonline.com/doi/abs/10.3200/JOEE.39.1.45-59.

[73] Schönfelder ML, Bogner FX. Two ways of acquiring environmental knowledge: by encountering living animals at a beehive and by observing bees via digital tools. Int J Sci Educ. 2017; 39(6):723–41. 10.1080/09500693.2017.1304670.

[74] Frantz CMP, Mayer FS. The importance of connection to nature in assessing environmental education programs. Stud Educ Eval [Internet]. 2014; 41:85–9. Available from: 10.1016/j.stueduc.2013.10.001.

[75] Liefländer AK, Fröhlich G, Bogner FX, Schultz PW. Promoting connectedness with nature through environmental education. Environ Educ Res. 2013; 19(3):370–384. 10.1080/13504622.2012.697545.

